# “Missed” Information: A Moral Failing that Erodes Efforts to Tackle the COVID-19 Pandemic

**DOI:** 10.3389/ijph.2021.1604667

**Published:** 2022-01-14

**Authors:** Leena Paakkari, Orkan Okan, Minna Torppa

**Affiliations:** ^1^ The Faculty of Sport and Health Sciences, University of Jyväskylä, Jyväskylä, Finland; ^2^ Faculty of Sports and Health Sciences, Technical University Munich, Munich, German; ^3^ Department of Teacher Education, University of Jyväskylä, Jyväskylä, Finland

**Keywords:** infodemic, health information, missed information, reading disabilities, coronavirus

Following the onset of the COVID-19 pandemic, a tsunami of information hit the internet–amounting to an information epidemic (infodemic) that itself developed into a global health problem [1]. It evolved especially in the social media, where people were offered an abundance of information, including information that might be over-complex, overwhelming, or in the worst case, totally false. False information can be spread to deliberately mislead people (disinformation) or without such an intent (misinformation), but both of these eventually create harm among information users [2]. In discussions on how to handle the infodemic, an underlying assumption has been that people do indeed have access to (i.e., have opportunities to reach, understand and use) information on the coronavirus, which then is their role to analyze and apply to the best of their ability. However, if one focuses on mis- and disinformation only, the problem of “missed information” can be overlooked in attempts to tackle infodemic. Here, the phrase “missed information” refers to valid and relevant information that people have not been able to access. People with reading difficulties is one of the hidden population groups vulnerable to miss important information on COVID-19.

To genuinely access and identify reliable information, people must have the skills “to navigate through multiple sources, to gauge, to prune, to compare and contrast information in order to build an integrated representation that makes sense out of what they read” [3], and further, to adapt the constituted message to their beliefs, needs, and goals. Today, these are seen as skills for all proficient readers, including children and adolescents [3]. Since information on the coronavirus is scattered, complex, and constantly increasing, the cognitive requirements for readers are tremendous.

Missed information is an obstacle to people’s empowerment, preventing them from the slowing the spread of the virus and protecting their health. It means that people are likely to become more vulnerable to the coronavirus and to the impacts of the infodemic (thus undermining trust in science and decreasing compliance with protective measures [1]). It also causes disparities between those who can and those who cannot access valid information. Indeed, the COVID-19 infodemic has underlined the need to secure information access for all. However, for instance people with reading difficulties—i.e., difficulties in identifying, processing, and comprehending written information—are in a particularly vulnerable situation, given that most relevant information is still presented in the written format, also on online.

In the first place, navigating the perpetual stream of information burdens cognitive processes that are known to be weakened in the case of reading difficulties, including vocabulary knowledge, linguistic comprehension, inference making, working memory, and attention focusing [4]. Secondly, due to the time and effort required to decode even brief texts, reading difficulties naturally lead to non-engagement with reading, especially if the texts are recognized as too demanding [4]. Thirdly, anxiety and depression often accompany reading difficulties [5], and these can lessen both the motivation and opportunity to cope with the demands set by a flood of information. Fourthly, reading difficulties are strongly associated with school dropout, and with lower educational status [6]. These factors pose a strong risk that individuals will miss important information from trustworthy sources, and will be vulnerable to the harmful effects of false information.

Reading difficulties affect hundreds of millions of people globally. Even when educational systems are fully in place, reading disability is the most common reason for special education programs, affecting 5–10% of people [7]. With regard to reading comprehension skills more broadly, the numbers are even higher. According to the PISA study [8], more than 20% of 15-year-olds do not reach the baseline reading level required in everyday live and secondary education. During the COVID-19 pandemic, an additional burden has resulted from school closures. These have had a particularly harmful impact on students with reading disabilities, who often need more support both to learn and to engage with learning. It is likely that due to school closures “the pandemic will push just under 100 million children below the proficiency threshold” in reading skills, in addition to the 483 million children already below the threshold [9]. When people with reading difficulties are added to persons who have other cognitive impairments, who do not receive information in their own language, who lack opportunities for schooling (e.g., due to school closures), and who are illiterate (incl., digitally illiterate), missed information can be seen as a matter of concern to over a billion people.

To secure access to valid information, the development of reading proficiency among adolescents and adults via education systems is not enough. It also requires a willingness to share institutional power over knowledge and knowing, and the creation of user-friendly information environments to meet the differing needs of individuals. Finally, especially in times of infodemic and pandemic, it is imperative to pay particular attention to the digital dimension, including both accessibility and literacy in the medium, and goes beyond purely technical solutions, constituting “a matter of political will and of moral obligation” [10]. Because it could be avoided, missed information indicates a moral failing on the part of society, eroding public health efforts to combat pandemics now and in the future.
